# Alcohol Consumption-Related Metabolites in Relation to Colorectal Cancer and Adenoma: Two Case-Control Studies Using Serum Biomarkers

**DOI:** 10.1371/journal.pone.0150962

**Published:** 2016-03-11

**Authors:** Jose Ramon Troche, Susan T. Mayne, Neal D. Freedman, Fatma M. Shebl, Kristin A. Guertin, Amanda J. Cross, Christian C. Abnet

**Affiliations:** 1 Yale School of Public Health, New Haven, Connecticut, United States of America; 2 Division of Cancer Epidemiology and Genetics, National Cancer Institute, National Institutes of Health, Department of Health and Human Services, Rockville, Maryland, United States of America; 3 Yale Cancer Center, New Haven, Connecticut, United States of America; 4 Department of Public Health Sciences, University of Virginia, Charlottesville, Virginia, United States of America; 5 Department of Epidemiology and Biostatistics, School of Public Health, Faculty of Medicine, Imperial College London, St. Mary’s Campus, Norfolk Place, London, United Kingdom; Centro Cardiologico Monzino, ITALY

## Abstract

Alcohol is a known carcinogen that may be associated with colorectal cancer. However, most epidemiologic studies assess alcoholic beverage consumption using self-reported data, leading to potential exposure misclassification. Biomarkers of alcohol consumption may provide an alternative, complementary approach that reduces misclassification and incorporates individual differences in alcohol metabolism. Therefore, we evaluated the relationship between previously identified alcohol consumption-related metabolites and colorectal cancer and adenoma using serum metabolomics data from two studies. Data on colorectal cancer were obtained from a nested case-control study of 502 US adults (252 cases, 250 controls) within the Prostate, Lung, Colorectal, and Ovarian Cancer Screening Trial. Data on colorectal adenoma were obtained from a case-control study of 197 US adults (120 cases, 77 controls) from the Navy Colon Adenoma Study. Unconditional multivariable logistic regression models were fit to calculate odds ratios (OR) and 95% confidence intervals (CI) for eight alcohol consumption-related metabolites identified in a previous analysis: ethyl glucuronide; 4-androstene-3beta,17beta-diol disulfate 1; 5-alpha-androstan-3beta,17beta-diol disulfate; 16-hydroxypalmitate; bilirubin (E,Z or Z,E); cyclo (-leu-pro); dihomo-linoleate (20:2n6); and palmitoleate (16:1n7). We found no clear association between these alcohol consumption-related metabolites and either endpoint. However, we did observe an inverse association between cyclo (-leu-pro) and colorectal adenoma that was only observed in the highest metabolite quantile (OR _4th vs. 1st Quantile_ = 0.30, 95% CI: 0.12–0.78; *P*-trend = 0.047), but no association for colorectal cancer. In conclusion, there were no adverse associations between alcohol consumption-related metabolites and colorectal cancer or adenoma.

## Introduction

Colorectal cancer ranks third in both cancer incidence and mortality in the US [1,2], with over 132,000 incident cases estimated for 2015 [2]. Colorectal cancer is more common in economically developed countries, while economically developing countries are seeing rapid increases in colorectal cancer incidence [3]. Some studies of colorectal cancer risk in migrants from lower-risk to higher-risk countries have found risk in migrants that approaches that of the host country [4,5], and that their US-born descendants have higher risks than foreign-born migrants [6]. These differing patterns may be due to modifiable lifestyle factors [3], including diet, physical inactivity, obesity, tobacco smoking, and alcohol consumption [7].

Alcoholic beverage consumption has been associated with colorectal cancer in men, and in many studies women [8], although the association is modest. The summary estimate of a 2010 meta-analysis showed an association with moderate (RR _2 to 3 drinks per day_ = 1.21, 95% CI: 1.13–1.28) and heavy drinking (RR _≥4 drinks per day_ = 1.52, 95% CI 1.27–1.81) [9]. The evidence is strongest for heavy drinkers [9–11] and does not appear to vary by alcoholic beverage type, implicating ethanol as a potential causative agent [11–14]. In observational studies, alcohol consumption has been associated with each anatomical site of colorectal cancer, including the proximal colon, distal colon, and rectum [10,11,14]. Less data are available in women. However, available data indicate similar findings as for men [8,11,14].

Alcoholic beverage consumption has also been associated with higher risk of adenomatous colorectal polyps [15–19]. Colorectal polyps can be broadly classified into two categories: hyperplastic polyps, which are typically benign, and adenomatous polyps (adenomas), which are precursor lesions to colorectal cancer. Although less than 10% of adenomas develop into colorectal cancer [1], most colorectal cancers develop from adenomas [1,20]. As such, these findings for alcohol with colorectal cancer and adenoma are complimentary.

Self-reported alcohol consumption data are generally considered to be accurate, and most epidemiologic studies rely on self-report to measure typical alcohol consumption [21]. However, self-reported data are subject to reporting error and, subsequently, exposure misclassification. Quantity-frequency measures also do not capture binge drinking and atypical drinking patterns, and this may underestimate total alcohol consumption [22]. In addition, self-reported alcohol consumption cannot account for individual differences in alcohol metabolism, such as ethnic differences observed in some Asian populations [23]. Therefore, the use of complementary measures of alcohol exposure in addition to self-report might be useful in more accurately estimating alcohol exposure [24]. Alcohol biomarkers, including small-molecule metabolites, could augment findings from self-report by adding objective measures of exposure and accounting for individual differences in internal dosing due to individual differences in metabolism. Metabolomics, the measurement of small molecules in biofluids, has been shown to be a useful method in epidemiologic investigations [25,26], with past studies successfully identifying biomarkers for tobacco smoking and diet, including alcohol [27,28].

In this study we evaluated the association between the following eight biomarkers of alcohol consumption and colorectal cancer and adenoma: ethyl glucuronide; 4-androstene-3beta,17beta-diol disulfate 1; 5-alpha-androstan-3beta,17beta-diol disulfate; 16-hydroxypalmitate; bilirubin (E,Z or Z,E); cyclo (-leu-pro); dihomo-linoleate (20:2n6); and palmitoleate (16:1n7). These metabolites were selected based on previous serum metabolomics data generated in the Prostate, Lung, Colorectal, and Ovarian Cancer Screening Trial (PLCO), where they were found to be associated with total alcoholic beverage consumption based on partial Pearson’s correlations after Bonferroni correction for multiple comparisons [27]. Using these biomarkers, we conducted a nested case-control study of colorectal cancer within the intervention arm of PLCO (252 cases, 250 controls), as well as a separate case-control study of colorectal adenoma (120 cases, 77 controls) in the Navy Colon Adenoma Study.

## Materials and Methods

### Study design and population

#### Prostate, Lung, Colorectal and Ovarian Cancer Screening Trial (PLCO)

PLCO is a population-based randomized controlled trial of the effects of screening on cancer mortality in the US [29,30]. Over 150,000 participants were enrolled between November 1993 and July 2001. Eligible participants were aged 55–74 years at the time of randomization, had no prior history of PLCO cancers, and had not had a sigmoidoscopy, colonoscopy, or barium enema within the three years prior to randomization. Written informed consent was obtained from all participants. The study was approved by the National Cancer Institute Institutional Review Board (protocol number OH97-C-N041).

Sigmoidoscopies were conducted at baseline and offered at the end of the third year prior to December 1998, and at the end of the fifth year beginning in October 1999. Participants with positive screen tests were referred to their physician for further evaluation.

Within the screening arm of PLCO, a nested case-control study selected participants who completed baseline risk factor and dietary questionnaires, consented to biospecimen use, had baseline serum and 6 months or greater of follow-up, had no self-reported history of cancer at baseline (excepting basal cell carcinoma), had no rare cancers during follow-up, and had no self-reported Crohn’s disease, ulcerative colitis, familial polyposis, Gardner syndrome, or colorectal polyps. Of these, 255 primary incident colorectal cancer cases and 254 controls were selected for our study. We further excluded 2 cases and 3 controls with 8 or greater missing responses on the dietary questionnaire and 1 case and 1 control with extreme caloric consumption (≤1% or ≥99%). Our final analytic sample for PLCO consisted of 252 cases and 250 controls.

Data on self-reported alcohol consumption were obtained from the baseline questionnaire. The following categories were chosen *a priori* based on multiples of a single alcoholic drink, which provided reasonable numbers of subjects in each category and which are similar to those in many previous studies of alcohol intake and disease: none, >0 to 1 drink per day, >1 to 3 drinks per day, and >3 drinks per day.

#### Navy Colon Adenoma Study

The Navy Colon Adenoma Study is a case-control study of colorectal adenomas which enrolled patients from the General Surgery and Gastroenterology Clinics at the National Naval Medical Institute in Bethesda, Maryland between April 1994 and September 1996. Eligible participants were aged 18–74 years, had no history of cancer (except non-melanoma skin cancer), and resided within 60 miles of Washington D.C. Written informed consent was obtained from all participants. The study was approved by the Institutional Review Boards at the National Cancer Institute and the National Naval Medical Center (protocol number OH93-NC-2004).

The study sample for the Navy Colon Adenoma Study has been described in detail elsewhere [31]. Briefly, 244 cases from a colonoscopy clinic register and 231 controls receiving routine flexible sigmoidoscopic screening gave informed consent and agreed to participate. We excluded 93 cases for having a history of previous adenomas, 2 cases and 3 controls with implausible dietary information, and 3 cases reporting familial adenomatous polyposis. Of these, 129 cases and 129 controls with height and weight data were selected for metabolic profiling in a previous analysis [32]. Cases and controls were not matched on fasting status, and preliminary analyses suggested that fasting status had substantial effects on metabolite concentrations. Therefore, we further excluded 6 cases and 51 controls who fasted before their blood draw. We then excluded 3 cases and 1 control with missing alcohol consumption data. Our final analytic sample for the Navy Colon Adenoma Study consisted of 120 cases and 77 controls.

Data on self-reported alcohol consumption were obtained during in-home personal interviews conducted approximately 60 to 90 days after endoscopic screening. We categorized alcohol consumption into the following categories: none, >0 to 1 drink per day, >1 to 3 drinks per day, and >3 drinks per day. Non-drinkers who reported ever consuming at least 12 alcoholic beverages in a year remained categorized as non-drinkers (*n* = 22).

### Outcome assessment

In PLCO, incident cancers were ascertained from the date of randomization (study entry date) through December 31, 2009. Sigmoidoscopies were conducted at baseline and offered at the end of the third year prior to December 1998, and at the end of the fifth year beginning in October 1999. Participants with positive screen tests were referred to their physician for further evaluation. The 255 first primary incident colorectal cancers diagnosed at least 6 months after baseline were selected as cases, as were 254 incidence-density sampled subjects who were alive and cancer free at the time the case was diagnosed and matched by age at randomization (5 year intervals), sex, race, randomization year, and season of blood draw.

In the Navy Colon Adenoma Study, colorectal adenoma cases were diagnosed by colonoscopy or routine flexible sigmoidoscopy and were histologically confirmed. Controls, selected from patients without colorectal adenomas at the time of sigmoidoscopy, were frequency matched (1:1) by age (±5 years) and sex to cases who were screened during the same time period.

### Metabolite assessment

In PLCO, non-fasting blood samples were collected at baseline. In the Navy Colon Adenoma Study, participants provided blood samples at follow-up clinic visits, and were not required to fast but many participants did. Serum metabolites were assayed by Metabolon Inc, as previously described [33,34]. The handling and metabolomics analysis of blood samples has also been described in detail elsewhere [26,27,32]. Samples were run in batches of 30 and blinded to case status. Cases and matched controls were placed consecutively within the batch. Samples were analyzed using ultra-high performance liquid-phase chromatography-mass spectrometry and gas chromatography-mass spectrometry. Metabolites were identified by comparing mass spectral peaks to a reference library. The relative mass spectral peak intensities for each metabolite were batch normalized (relative peak intensity value divided by batch median), log-transformed (natural log), and centered (mean≈0, standard deviation≈1). The minimum observed value was imputed for measurements which failed to reach the detection threshold and the distributions were centered. Assay precision has previously been studied in detail [26].

A previous serum metabolomics study of PLCO tested correlations between diet and metabolites using partial Pearson’s correlations adjusted for age, sex, smoking status (current smokers vs. former/never smokers), and total energy intake (kcal per day) [27]. After Bonferroni correction for multiple comparisons, eight metabolites were significantly associated with total alcohol: ethyl glucuronide; 4-androstene-3beta,17beta-diol disulfate 1; 5-alpha-androstan-3beta,17beta-diol disulfate; 16-hydroxypalmitate; bilirubin (E,Z or Z,E); cyclo (-leu-pro); dihomo-linoleate (20:2n6); and palmitoleate (16:1n7), and these were used in our study.

### Statistical analysis

We used multivariable unconditional logistic regression to estimate odds ratios (OR) and 95% confidence intervals (CI) for the relationship between self-reported alcohol and the eight alcohol consumption-related metabolites and colorectal cancer (PLCO) and adenoma (Navy Colon Adenoma Study). The analyses were conducted separately in each study due to differences in study design, study populations, and data collection methods. All models were adjusted for age at dietary questionnaire (PLCO) or interview (Navy Colon Adenoma Study), sex, and tobacco use (never tobacco user, former tobacco user, current tobacco user). Tobacco use included cigarettes, cigars, and pipes.

The eight alcohol consumption-related metabolites were not normally distributed, in part because values below the detection threshold were assigned the minimum observed value. Five of the eight metabolites (4-androstene-3beta,17beta-diol disulfate 1; 5-alpha-androstan-3beta,17beta-diol disulfate; 16-hydroxypalmitate; dihomo-linoleate (20:2n6); palmitoleate (16:1n7)) were categorized based on the quartile cutpoints among controls. The remaining three metabolites could not be categorized exactly by quartiles due to the large number of imputed values. Ethyl glucuronide was categorized as a binary variable (detected, not detected). Bilirubin (E,Z or Z,E) was categorized by quartile cutpoints in the Navy Colon Adenoma Study, but in PLCO was categorized into the following percentile groups (≤27.5, >27.5 to 50.0, >50.0 to 75.0, >75.0^th^ percentile). Cyclo (-leu-pro) was categorized differently in PLCO (≤51.4, >51.4 to 75.0, >75.0^th^ percentile) than in the Navy Colon Adenoma Study (≤25.4, >25.4 to 50.0, >50.0 to 75.0, >75.0^th^ percentile).

We also examined bivariate associations between self-reported alcohol consumption and the eight alcohol consumption-related metabolites previously found to be significantly correlated with total alcohol consumption based on partial Pearson’s correlations adjusted for age, sex, smoking status, and total energy intake [27]. To account for the non-normal distribution of the metabolites, we categorized the metabolites based on approximate quartile cutpoints and then retested their association with alcohol using Fisher’s exact tests. Therefore, the tests we conducted had lower power than the analysis from which our metabolites were identified.

Because we had eight metabolites identified by their independent correlation with self-reported alcoholic beverage consumption, we explored their independence and completed a data reduction for use as a secondary exposure in the association analysis. We used a matrix of Pearson’s correlation coefficients to assess correlation among the metabolites and to visually assess whether a subset of metabolites might be used in substitution. A principal component analysis using varimax rotation was employed as a method to reduce the number of exposure variables. We retained components based on the eigenvalue-one criterion [35] and those which cumulatively explained greater than 70% of the variance in the covariance matrix of the eight alcohol consumption-related metabolites. These meaningful components were then extracted, scaled by dividing by half the interquartile range, and fit together in a full model, adjusting for age, sex, and tobacco use.

All analyses were conducted using SAS Version 9.2 TS2M3 (SAS Inc., Cary, North Carolina, USA).

## Results

The sample was predominantly male in both PLCO (cases: 56.4%, controls: 55.6%) and the Navy Colon Adenoma Study (cases: 75.8%, controls: 74.0%) (Table 1). The mean age was higher in PLCO (cases: 64.3 years, controls: 64.3 years) than the Navy Colon Adenoma Study (cases: 57.4 years, controls: 57.6 years). The proportion of never tobacco users was similar between PLCO (cases: 38.0%, controls: 42.0%) and the Navy Colon Adenoma Study (cases: 46.7%, controls: 42.9%), whereas the proportion of current smokers was higher in PLCO (cases: 47.6%, controls: 49.2%) than in the Navy Colon Adenoma Study (cases: 10.0%, controls: 9.1%).

**Table 1:** Selected Characteristics in 502 US Adults in the Prostate, Lung, Colorectal, and Ovarian Cancer Screening Trial (PLCO) and 197 US Adults in the Navy Colon Adenoma Study.

Characteristic	PLCO		Navy Colon Adenoma Study	
	Case	Control	Case	Control
	N (Percent)	N (Percent)	N (Percent)	N (Percent)
*Analytic Sample*	252 (100.0)	250 (100.0)	120 (100.0)	77 (100.0)
*Age (Years)*, *Mean (SD)*	64.3 (5.1)	64.3 (5.1)	57.4 (9.1)	57.6 (8.2)
*Sex*				
Female	110 (43.7)	111 (44.4)	29 (24.2)	20 (26.0)
Male	142 (56.4)	139 (55.6)	91 (75.8)	57 (74.0)
*Tobacco Use*				
Never Used Tobacco	95 (38.0)	105 (42.0)	56 (46.7)	33 (42.9)
Former Tobacco User	36 (14.4)	22 (8.8)	52 (43.3)	37 (48.1)
Current Tobacco User	119 (47.6)	123 (49.2)	12 (10.0)	7 (9.1)
*Education*				
≤High School or Equivalent	83 (32.9)	84 (33.7)	19 (15.8)	4 (5.3)
1 to 3 years College/Vocational	84 (33.3)	75 (30.1)	24 (20.0)	14 (18.4)
College Graduate	42 (16.7)	46 (18.5)	21 (17.5)	17 (22.4)
Post Graduate	43 (17.1)	44 (17.7)	56 (46.7)	41 (54.0)
*Race/Ethnicity*				
White, Non-Hispanic	225 (89.3)	223 (89.2)	102 (85.0)	66 (85.7)
Black, Non-Hispanic	13 (5.2)	13 (5.2)	13 (10.8)	6 (7.8)
Hispanic	3 (1.2)	5 (2.0)	0 (0.0)	2 (2.6)
Other	11 (4.4)	9 (3.6)	5 (4.2)	3 (3.9)
*Family History of Colon Cancer*				
No	210 (84.0)	217 (87.5)	100 (83.3)	67 (88.2)
Yes	33 (13.2)	24 (9.7)	20 (16.7)	9 (11.8)
Possibly	7 (2.8)	7 (2.8)	-	-
*Physical Activity (Hours/Week)*				
0 to <1	75 (29.8)	78 (31.5)	15 (12.5)	6 (7.8)
1	31 (12.3)	25 (10.1)	6 (5.0)	2 (2.6)
2	47 (18.7)	36 (14.5)	5 (4.2)	4 (5.2)
3	34 (13.5)	34 (13.7)	8 (6.7)	4 (5.2)
≥4	65 (25.8)	75 (30.2)	86 (71.7)	61 (79.2)
*Body Mass Index*, *Median (IQR)*	27.4 (6.6)	26.5 (5.5)	26.5 (4.4)	26.4 (6.0)
*Calories*, *Median (IQR)*	1894.6 (1118.7)	1951.5 (954.8)	1487.6 (720.9)	1535.8 (729.5)
*Alcohol Consumption (Drinks/Day)*				
None	54 (21.4)	49 (19.6)	29 (24.2)	15 (19.5)
>0 to 1	139 (55.2)	127 (50.8)	39 (32.5)	27 (35.1)
>1 to 3	32 (12.7)	47 (18.8)	35 (29.2)	25 (32.5)
>3	27 (10.7)	27 (10.8)	17 (14.2)	10 (13.0)

SD Standard Deviation, IQR Interquartile Range.

We examined bivariate associations between demographic variables and self-reported alcohol consumption among controls (Table 2). Alcohol consumption was common among controls (PLCO: 80.4%, Navy Colon Adenoma Study: 80.5%) (Table 2). Across the two studies, a similar proportion of control drinkers consumed >3 drinks per day (PLCO: 10.8%, Navy Colon Adenoma Study: 13.0%). Consuming >3 drinks per day was also more common in males than females (PLCO: 18.0% vs. 1.8%, Navy Colon Adenoma Study: 15.8% vs. 5.0%). Among controls, compared with non-drinkers a higher proportion of alcohol drinkers were ever smokers (PLCO: 61.2% vs. 44.9%, Navy Colon Adenoma Study: 61.3% vs. 40.0%).

**Table 2:** Selected Characteristics Among Controls by Categories of Alcohol Consumption in 250 US Adults (PLCO) and 77 US Adults.

Characteristic	PLCO					Navy Colon Adenoma Study				
	Alcohol Category (Drinks/Day), N^a^ (Percent)				*P*-Value^b^	Alcohol Category (Drinks/Day), N^a^ (Percent)				*P*-Value^b^
	None	>0 to 1	>1 to 3	>3		None	>0 to 1	>1 to 3	>3	
Analytic Sample	49 (19.6)	127 (50.8)	47 (18.8)	27 (10.8)	-	15 (19.5)	27 (35.1)	25 (32.5)	10 (13.0)	-
Age (Years), Mean (SD)	64.2 (5.7)	64.0 (4.9)	65.0 (5.1)	64.9 (5.1)	0.78	56.8 (7.9)	55.3 (9.0)	58.8 (6.8)	62.2 (8.2)	0.14
Sex										
Male	26 (18.7)	56 (40.3)	32 (23.0)	25 (18.0)	<0.0010	8 (14.0)	19 (33.3)	21 (36.8)	9 (15.8)	0.12
Female	23 (20.7)	71 (64.0)	15 (13.5)	2 (1.8)		7 (35.0)	8 (40.0)	4 (20.0)	1 (5.0)	
Tobacco Use										
Never Used Tobacco	27 (25.7)	59 (56.2)	15 (14.3)	4 (3.8)	0.0010	9 (27.3)	12 (36.4)	11 (33.3)	1 (3.0)	0.16
Former Tobacco User	21 (17.1)	59 (48.0)	23 (18.7)	20 (16.3)		5 (13.5)	12 (32.4)	11 (29.7)	9 (24.3)	
Current Tobacco User	1 (4.6)	9 (40.9)	9 (40.9)	3 (13.6)		1 (14.3)	3 (42.9)	3 (42.9)	0 (0.0)	
Education										
≤High School or Equivalent	23 (27.4)	42 (50.0)	10 (11.9)	9 (10.7)	0.055	1 (25.0)	2 (50.0)	0 (0.0)	1 (25.0)	0.18
1 to 3 years College/Vocational	16 (21.3)	38 (50.7)	11 (14.7)	10 (13.3)		5 (35.7)	3 (21.4)	4 (28.6)	2 (14.3)	
College Graduate	4 (8.7)	22 (47.8)	15 (32.6)	5 (10.9)		5 (29.4)	6 (35.3)	6 (35.3)	0 (0.0)	
Post-Graduate	5 (11.4)	25 (56.8)	11 (25.0)	3 (6.8)		4 (9.8)	16 (39.0)	14 (34.2)	7 (17.1)	
Race/Ethnicity										
White, Non-Hispanic	39 (17.5)	117 (52.5)	43 (19.3)	24 (10.8)	0.31	12 (18.2)	21 (31.8)	24 (36.4)	9 (13.6)	0.60
Black, Non-Hispanic	6 (46.2)	4 (30.8)	2 (15.4)	1 (7.7)		2 (33.3)	2 (33.3)	1 (16.7)	1 (16.7)	
Hispanic	1 (20.0)	3 (60.0)	1 (20.0)	0 (0.0)		0 (0.0)	2 (100.0)	0 (0.0)	0 (0.0)	
Other	3 (33.3)	3 (33.3)	1 (11.1)	2 (22.2)		1 (33.3)	2 (66.7)	0 (0.0)	0 (0.0)	
Family History of Colon Cancer										
No	41 (18.9)	109 (50.2)	42 (19.4)	25 (11.5)	0.88	13 (19.4)	23 (34.3)	22 (32.8)	9 (13.4)	1.00
Yes	5 (20.8)	13 (54.2)	4 (16.7)	2 (8.3)		2 (22.2)	3 (33.3)	3 (33.3)	1 (11.1)	
Uncertain	3 (42.9)	3 (42.9)	1 (14.3)	0 (0.0)		-	-	-	-	
Physical Activity (Hours/Week)										
None	18 (23.1)	38 (48.7)	14 (18.0)	8 (10.3)	0.64	3 (50.0)	0 (0.0)	2 (33.3)	1 (16.7)	0.22
1	7 (28.0)	10 (40.0)	4 (16.0)	4 (16.0)		0 (0.0)	0 (0.0)	2 (100.0)	0 (0.0)	
2	9 (25.0)	20 (55.6)	4 (11.1)	3 (8.3)		1 (25.0)	3 (75.0)	0 (0.0)	0 (0.0)	
3	3 (8.8)	21 (61.8)	7 (20.6)	3 (8.8)		1 (25.0)	1 (25.0)	2 (50.0)	0 (0.0)	
≥4	11 (14.7)	38 (50.7)	17 (22.7)	9 (12.0)		10 (16.4)	23 (37.7)	19 (31.2)	9 (14.8)	
Body Mass Index, Median (IQR)	26.6 (5.0)	26.5 (6.3)	25.1 (5.3)	27.2 (4.3)	0.43	26.5 (7.5)	25.8 (5.4)	25.8 (4.3)	29.0 (9.3)	0.70
Calories, Median (IQR)	1,614 (984)	1,873 (835)	2,121 (831)	2,339 (972)	<0.0010	1,091 (835)	1,388 (674)	1,553 (467)	2,786 (1,088)	0.0010

^a^ May not sum to total due to missing values.

^b^ Fisher’s exact test (categorical) or Wilcoxon rank sum (continuous).

We next examined bivariate associations between self-reported alcohol consumption and the eight alcohol consumption-related metabolites. In PLCO, only five of the eight metabolites were significantly positively associated with self-reported alcohol consumption: ethyl glucuronide (*P* < 0.0010); 4-androstene-3beta,17beta-diol disulfate 1 (*P* < 0.0010); 5-alpha-androstan-3beta,17beta-diol disulfate (*P* < 0.0010); bilirubin (E,Z or Z,E) (*P* = 0.0024); and cyclo (-leu-pro) (*P* = 0.045) (S1 Table). For each of these five metabolites, a greater proportion of non-drinkers were distributed in the lowest metabolite quantile, while a higher proportion of those who drank >3 drinks per day were distributed in the highest metabolite quantile. In the Navy Colon Adenoma Study, there were only 77 controls, which limited power. None of the eight metabolites were significantly associated with self-reported alcohol, however a similar pattern to PLCO was evident for the following alcohol consumption-related metabolites: 4-androstene-3beta,17beta-diol disulfate 1 (*P* = 0.44); 5-alpha-androstan-3beta,17beta-diol disulfate (*P* = 0.079); bilirubin (E,Z or Z,E) (*P* = 0.65); and cyclo (-leu-pro) (*P* = 0.12). For these four metabolites, a greater proportion of non-drinkers were distributed in the lowest metabolite quantile, while a greater proportion of those consuming >1 to 3 drinks per day were distributed in the highest metabolite quantile.

Because these novel biomarkers are all correlated with self-reported alcoholic beverage consumption, we assessed their independence and to carry out a data reduction to independent vectors of exposure. We evaluated the Pearson’s correlation coefficients among the eight alcohol consumption-related metabolites in a correlation matrix (S2 Table), with absolute values ranging from 0.028 to 0.88 in PLCO and 0.026 to 0.89 in the Navy Colon Adenoma Study. In both PLCO and the Navy Colon Adenoma Study, there were two distinct groupings of metabolites with correlation coefficients greater than 0.50: 1) 16-hydroxypalmitate; dihomo-linoleate (20:2n6); palmitoleate (16:1n7); and 2) 4-androstene-3beta,17beta-diol disulfate 1 and 5-alpha-androstan-3beta,17beta-diol disulfate. A subsequent principal component analysis identified three meaningful components. In PLCO, these components cumulatively explained 74.2% of the variance in the covariance matrix of the eight alcohol consumption-related metabolites. Individually, components 1, 2, and 3 explained 38.3%, 23.2%, and 12.7% of the variance, respectively. Similarly, in the Navy Colon Adenoma Study the components cumulatively explained 75.9% of the variance, with components 1, 2, and 3 respectively explaining 40.2%, 22.5%, and 13.1% of the variance.

Next, we examined associations with our disease endpoints. In fully adjusted models, we found no association between self-reported typical alcohol consumption and colorectal cancer (*P*-trend = 0.25; Table 3) or colorectal adenoma (*P*-trend = 0.96; Table 4). Likewise, none of the eight alcohol consumption-related metabolites were significantly associated with colorectal cancer, and seven were not significantly associated with colorectal adenoma. However, one metabolite, cyclo (-leu-pro), was significantly associated with colorectal adenoma (*P*-trend = 0.047). Compared with relative metabolite levels of cyclo (-leu-pro) among participants in the lowest quantile, those in the in the second and third quantiles were not significantly associated with colorectal adenoma (OR _2nd vs. 1st Quantile_ = 1.11, 95% CI: 0.51–2.44; OR _3rd vs. 1st Quantile_ = 1.05, 95% CI: 0.47–2.38), whereas peak intensities in the highest quantile were significantly inversely associated with colorectal adenoma (OR _4th vs. 1st Quantile_ = 0.30, 95% CI: 0.12–0.78).

**Table 3:** Adjusted Odds Ratios and 95% Confidence Intervals for the Association Between Self-Reported Alcohol Consumption/Alcohol Consumption-Related Metabolites and Colorectal Cancer in 502 US Adults (PLCO).

Metabolite	Categories				Global P-Value	*P*-trend^a^
**Self-Reported Alcohol Consumption**	**Alcohol Category (Drinks/Day)**					
	**None**	**>0 to 1**	**>1 to 3**	**>3**	3 df test	
No. Cases/Controls	54/49	139/127	32/47	27/27	0.20	0.25
OR^†^ (95% CI)	1.00 (ref)	0.97 (0.61–1.54)	0.56 (0.31–1.04)	0.81 (0.41–1.61)		
**Ethyl Glucuronide**	**≤91.43 percentile**	**>91.43 percentile**	-	-	1 df test	
No. Cases/Controls	224/235	28/15	-	-	0.059	0.064
OR^†^ (95% CI)	1.00 (ref)	1.87 (0.96–3.62)	-	-		
**4-androstene-3beta,17beta-diol disulfate 1**	**≤25 percentile**	**>25 to 50 percentile**	**>50 to 75 percentile**	**>75 percentile**	3 df test	
No. Cases/Controls	64/63	64/62	57/63	67/62	0.94	0.86
OR^†^ (95% CI)	1.00 (ref)	0.98 (0.58–1.64)	0.86 (0.50–1.49)	0.98 (0.55–1.73)		
**5-alpha-androstan-3beta,17beta-diol disulfate**	**≤25 percentile**	**>25 to 50 percentile**	**>50 to 75 percentile**	**>75 percentile**	3 df test	
No. Cases/Controls	63/63	64/62	60/63	65/62	0.96	0.67
OR^†^ (95% CI)	1.00 (ref)	0.95 (0.55–1.63)	0.85 (0.44–1.63)	0.91 (0.46–1.81)		
**16-Hydroxypalmitate**	**≤25 percentile**	**>25 to 50 percentile**	**>50 to 75 percentile**	**>75 percentile**	3 df test	
No. Cases/Controls	74/62	70/63	53/62	55/63	0.48	0.14
OR^†^ (95% CI)	1.00 (ref)	0.92 (0.56–1.49)	0.72 (0.44–1.20)	0.73 (0.44–1.20)		
**Bilirubin (E,Z or Z,E)**	**≤27.49 percentile**	**>27.49 to 50 percentile**	**>50 to 75 percentile**	**>75 percentile**	3 df test	
No. Cases/Controls	74/69	45/56	79/63	54/62	0.33	0.81
OR^†^ (95% CI)	1.00 (ref)	0.77 (0.46–1.29)	1.19 (0.75–1.91)	0.84 (0.51–1.39)		
**Cyclo (-Leu-Pro)**	**≤51.39 percentile**	**>51.39 to 75 percentile**	**>75 percentile**	-	2 df test	
No. Cases/Controls	138/129	60/59	54/62	-	0.47	0.26
OR^†^ (95% CI)	1.00 (ref)	0.91 (0.59–1.42)	0.75 (0.48–1.18)	-		
**Dihomo-linoleate (20:2n6)**	**≤25 percentile**	**>25 to 50 percentile**	**>50 to 75 percentile**	**>75 percentile**	3 df test	
No. Cases/Controls	72/62	59/60	68/66	53/62	0.71	0.37
OR^†^ (95% CI)	1.00 (ref)	0.88 (0.53–1.45)	0.97 (0.59–1.58)	0.76 (0.46–1.26)		
**Palmitoleate (16:1n7)**	**≤25 percentile**	**>25 to 50 percentile**	**>50 to 75 percentile**	**>75 percentile**	3 df test	
No. Cases/Controls	69/62	52/56	77/69	54/63	0.63	0.52
OR^†^ (95% CI)	1.00 (ref)	0.87 (0.52–1.45)	1.06 (0.66–1.72)	0.79 (0.47–1.32)		

Abbreviations: CI, Confidence Interval; OR, Odds Ratio.

^†^ Adjusted for age (continuous), sex (binary: female vs. male), and tobacco smoking (categorical: current tobacco user, former tobacco user, never tobacco user, missing tobacco data).

^a^ Trend was tested using an ordinal variable containing the median value of each alcohol category (self-report) or the median value of the metabolite categories (alcohol consumption-related metabolites).

**Table 4:** Adjusted Odds Ratios and 95% Confidence Intervals for the Association Between Self-Reported Alcohol Consumption/Alcohol Consumption-Related Metabolites and Colorectal Adenoma in 197 US Adults (Navy Colon Adenoma Study).

Metabolite	Categories				Global P-Value	*P*-trend^a^
**Self-Reported Alcohol Consumption**	**Alcohol Category (Drinks/Day)**					
	**None**	**>0 to 1**	**>1 to 3**	**>3**	3 df test	
No. Cases/Controls	29/15	39/27	35/25	17/10	0.86	0.96
OR^†^ (95% CI)	1.00 (ref)	0.75 (0.33–1.72)	0.71 (0.30–1.68)	0.86 (0.29–2.60)		
**Ethyl Glucuronide**	**≤72.59 percentile**	**>72.59 percentile**	-	-	1 df test	
No. Cases/Controls	98/56	22/21	-	-	0.15	0.15
OR^†^ (95% CI)	1.00 (ref)	0.58 (0.28–1.21)	-	-		
**4-androstene-3beta,17beta-diol disulfate 1**	**≤25 percentile**	**>25 to 50 percentile**	**>50 to 75 percentile**	**>75 percentile**	3 df test	
No. Cases/Controls	34/20	26/19	23/19	37/19	0.62	0.70
OR^†^ (95% CI)	1.00 (ref)	0.80 (0.35–1.80)	0.67 (0.29–1.57)	1.14 (0.49–2.62)		
**5-alpha-androstan-3beta,17beta-diol disulfate**	**≤25 percentile**	**>25 to 50 percentile**	**>50 to 75 percentile**	**>75 percentile**	3 df test	
No. Cases/Controls	35/19	33/20	28/19	24/19	0.68	0.26
OR^†^ (95% CI)	1.00 (ref)	0.79 (0.34–1.86)	0.66 (0.27–1.62)	0.57 (0.22–1.48)		
**16-Hydroxypalmitate**	**≤25 percentile**	**>25 to 50 percentile**	**>50 to 75 percentile**	**>75 percentile**	3 df test	
No. Cases/Controls	37/19	26/20	32/19	25/19	0.69	0.48
OR^†^ (95% CI)	1.00 (ref)	0.66 (0.29–1.49)	0.87 (0.39–1.96)	0.67 (0.29–1.52)		
**Bilirubin (E,Z or Z,E)**	**≤25 percentile**	**>25 to 50 percentile**	**>50 to 75 percentile**	**>75 percentile**	3 df test	
No. Cases/Controls	30/19	24/19	42/20	24/19	0.51	0.79
OR^†^ (95% CI)	1.00 (ref)	0.82 (0.35–1.90)	1.42 (0.63–3.19)	0.85 (0.36–1.99)		
**Cyclo (-Leu-Pro)**	**≤25.38 percentile**	**>25.38 to 50 percentile**	**>50 to 75 percentile**	**>75 percentile**	3 df test	
No. Cases/Controls	35/19	40/20	34/18	11/20	0.022	0.047
OR^†^ (95% CI)	1.00 (ref)	1.11 (0.51–2.44)	1.05 (0.47–2.38)	0.30 (0.12–0.78)		
**Dihomo-linoleate (20:2n6)**	**≤25 percentile**	**>25 to 50 percentile**	**>50 to 75 percentile**	**>75 percentile**	3 df test	
No. Cases/Controls	33/19	34/19	32/20	21/19	0.69	0.25
OR^†^ (95% CI)	1.00 (ref)	1.01 (0.45–2.27)	0.91 (0.41–2.02)	0.64 (0.27–1.48)		
**Palmitoleate (16:1n7)**	**≤25 percentile**	**>25 to 50 percentile**	**>50 to 75 percentile**	**>75 percentile**	3 df test	
No. Cases/Controls	38/19	32/19	26/19	24/20	0.65	0.24
OR^†^ (95% CI)	1.00 (ref)	0.86 (0.39–1.90)	0.70 (0.31–1.58)	0.61 (0.27–1.39)		

Abbreviations: CI, Confidence Interval; OR, Odds Ratio.

^†^ Adjusted for age (continuous), sex (binary: female vs. male), and tobacco smoking (categorical: current tobacco user, former tobacco user, never tobacco user, missing tobacco data).

^a^ Trend was tested using an ordinal variable containing the median value of each alcohol category (self-report) or the median value of the metabolite categories (alcohol consumption-related metabolites).

Finally, we tested for associations with disease outcome using the uncorrelated eigenvectors derived from the data reduction analysis. In PLCO, none of the components were associated with colorectal cancer (S3 Table). In the Navy Colon Adenoma Study, components 1 and 2 were not significantly associated with colorectal adenoma. However, component 3, which was loaded heavily from cyclo (-leu-pro), was significantly associated with lower risk for colorectal adenoma (OR _Component 3_ = 0.24, 95% CI: 0.06–0.94).

## Discussion

In our investigation of alcohol exposure using novel serum biomarkers and risk of colorectal cancer or colorectal adenoma, we observed little evidence for associations. Our null findings using these novel serum biomarkers differ from the majority of existing evidence for self-reported alcoholic beverage use, but are in line with some previous studies that also reported no significant association between total self-reported alcohol consumption and colorectal cancer [36–38] or colorectal adenoma [39–41].

We found one nominally significant inverse association between higher cyclo (-leu-pro) and lower risk of colorectal adenoma in the Navy Colon Adenoma Study. In PLCO, about half of control subjects had values below the limit of detection for this metabolite which limited our ability to test for differences across a spectrum of exposure, but those subjects in the highest category were at non-significantly reduced risk of colorectal cancer. Cyclo (-leu-pro), a cyclic dipeptide of leucine and proline, is a byproduct of alcoholic beverage fermentation [42], but the apparent association with colorectal adenoma should be interpreted with caution given the modest size of the study and multiple comparisons in the analysis.

Furthermore, because these novel biomarkers must have some degree of correlation, given that they were selected based on their independent correlation with self-reported alcoholic beverage consumption, we completed a correlation analysis and principal component analysis. This analysis showed that there were three independent vectors of information among the eight metabolites that could represent different aspects of alcoholic beverage consumption and metabolism. These findings could form the basis of future work to better define metabolic profiles associated with alcoholic beverage consumption. In the association analysis with these vectors, the one nominally significant association we reported for colorectal adenoma was also evident in the vector most weighted by cyclo (-leu-pro), but the data reduction did not provide any other additional insight in our data.

Our investigation of alcohol consumption-related metabolites used an innovative approach in evaluating the relationship between alcohol consumption and colorectal cancer and adenoma. The existing evidence is based primarily on self-reported alcohol consumption and the strengths of the reported associations are modest [43–46]. Biomarkers of alcohol consumption can potentially provide certain advantages over self-report by avoiding reporting error, reducing misclassification, and encompassing individual differences in internal dosing due to individual differences in metabolism. For example, in a previous study in PLCO, tobacco metabolites were found to have a stronger association with colorectal cancer risk than self-reported tobacco exposure [28].

In addition to the use of novel biomarkers of alcohol exposure, our investigation has other strengths. First, the study in PLCO was nested within a large prospective study which yielded 252 cases of colorectal cancer with baseline biomarker data. Second, both studies included detailed information on potential confounders. Our study also included some limitations. First, the metabolomics platform used for our study provided only relative peak intensities, rather than metabolite concentrations. Second, the half-lives of the eight alcohol consumption-related metabolites used in our analysis are unknown, and the time frame of alcohol exposure they capture is unclear. We lack data on when alcohol was last consumed prior to sample collection, and we are unsure how these alcohol consumption-related metabolites are influenced by lifetime drinking, atypical drinking patterns, and binge drinking. However, future work is in progress to examine the effects of controlled alcohol ingestion in the Women’s Alcohol Study, which has been described previously [47]. Third, in PLCO, only five of the eight metabolites were significantly positively associated with self-reported alcohol consumption, and none were significant in the Navy Colon Adenoma Study. Fourth, there were relatively few heavy drinkers, so we were not powered to evaluate associations at higher levels of alcoholic beverage consumption. Fifth, alcohol may have different associations with different subtypes of colorectal cancer, but we did not examine these relationships in this study.

In summary, we found no clear evidence for an association between alcohol consumption-related metabolites and either colorectal cancer or colorectal adenoma. Our data should not be interpreted as a refutation of studies using self-reported data because these novel biomarkers represent complementary, not competing, methods of assessing alcoholic beverage consumption. A potential inverse association between cyclo (-leu-pro) and adenoma that was only observed in the highest metabolite quantile should be evaluated in other data sets.

## Supporting Information

S1 Table Alcohol Consumption-Related Metabolites Among Controls by Categories of Alcohol Consumption in 250 US Adults (PLCO) and 77 US Adults (Navy Colon Adenoma Study). * May not sum to total due to missing values.
(DOCX)

S2 Table Pearson’s Correlation Matrix for Alcohol Consumption-Related Metabolites in 502 US Adults (PLCO) and 197 US Adults (Navy Colon Adenoma Study). (DOCX)

S3 Table Adjusted Odds Ratios and 95% Confidence Intervals for the Association Between Principal Components Extracted From Principal Component Analysis and fit in a Logistic Regression Model for Colorectal Cancer in 502 US Adults (PLCO) and Colorectal Adenoma in 197 US Adults (Navy Colon Adenoma Study). Abbreviations: CI, Confidence Interval; OR, Odds Ratio. ^†^ Adjusted for age (continuous), sex (binary: female vs. male), and tobacco smoking (categorical: current tobacco user, former tobacco user, never tobacco user, missing tobacco data). ^a^ Principal components were scaled by dividing by half the interquartile range and were then analyzed together in an unconditional multivariable logistic regression model, adjusting for age, sex, and tobacco use.
(DOCX)

## Abbreviations

CI: Confidence Interval
OR: Odds Ratio
PLCO: Prostate, Lung, Colorectal, and Ovarian Cancer Screening Trial
