# Supplementary material for: Alcohol Consumption-Related Metabolites in Relation to Colorectal Cancer and Adenoma: Two Case-Control Studies Using Serum Biomarkers
Source: PLoS One. 2016 Mar 11;11(3):e0150962. doi: 10.1371/journal.pone.0150962 (PMC4788441; doi:10.1371/journal.pone.0150962)
Supplement: S1 Table — * May not sum to total due to missing values. (DOCX) [file pone.0150962.s001.docx]

| **S1 Table. Alcohol Consumption-Related Metabolites Among Controls by Categories of Alcohol Consumption in 250 US Adults (PLCO) and 77 US Adults (Navy Colon Adenoma Study)** | | | | | | | | | | | |
| --- | --- | --- | --- | --- | --- | --- | --- | --- | --- | --- | --- |
| Characteristic | PLCO | | | | |  | Navy Colon Adenoma Study | | | | |
|  | Alcohol Category (Drinks/Day), N* (Percent) | | | | $\chi^{2}$ *P*-Value ^a^ |  | Alcohol Category (Drinks/Day), N* (Percent) | | | | $\chi^{2}$ *P*-Value ^a^ |
|  | None | >0 to 1 | >1 to 3 | >3 |  |  | None | >0 to 1 | >1 to 3 | >3 |  |
| **Ethyl Glucuronide** |  |  |  |  |  | **Ethyl Glucuronide** |  |  |  |  |  |
| ≤91.43 percentile | 48 (98.0) | 124 (97.6) | 42 (89.4) | 21 (77.8) | <0.0010 | ≤72.59 percentile | 14 (93.3) | 20 (74.1) | 14 (56.0) | 8 (80.0) | 0.070 |
| >91.43 percentile | 1 (2.0) | 3 (2.4) | 5 (10.6) | 6 (22.2) |  | >72.59 percentile | 1 (6.7) | 7 (25.9) | 11 (44.0) | 2 (20.0) |  |
| **4-androstene-3beta,17beta-diol disulfate 1** |  |  |  |  |  | **4-androstene-3beta,17beta-diol disulfate 1** |  |  |  |  |  |
| ≤25 percentile | 18 (36.7) | 36 (28.4) | 7 (14.9) | 2 (7.4) | <0.0010 | ≤25 percentile | 5 (33.3) | 6 (22.2) | 4 (16.0) | 5 (50.0) | 0.44 |
| >25 to 50 percentile | 13 (26.5) | 36 (28.4) | 8 (17.0) | 5 (18.5) |  | >25 to 50 percentile | 3 (20.0) | 7 (25.9) | 6 (24.0) | 3 (30.0) |  |
| >50 to 75 percentile | 9 (18.4) | 34 (26.8) | 17 (36.2) | 3 (11.1) |  | >50 to 75 percentile | 5 (33.3) | 8 (29.6) | 5 (20.0) | 1 (10.0) |  |
| >75 percentile | 9 (18.4) | 21 (16.5) | 15 (31.9) | 17 (63.0) |  | >75 percentile | 2 (13.3) | 6 (22.2) | 10 (40.0) | 1 (10.0) |  |
| **5-alpha-androstan-3beta,17beta-diol disulfate** |  |  |  |  |  | **5-alpha-androstan-3beta,17beta-diol disulfate** |  |  |  |  |  |
| ≤25 percentile | 18 (36.7) | 36 (28.4) | 8 (17.0) | 1 (3.7) | <0.0010 | ≤25 percentile | 4 (26.7) | 7 (25.9) | 5 (20.0) | 3 (30.0) | 0.079 |
| >25 to 50 percentile | 11 (22.5) | 40 (31.5) | 8 (17.0) | 3 (11.1) |  | >25 to 50 percentile | 8 (53.3) | 6 (22.2) | 3 (12.0) | 3 (30.0) |  |
| >50 to 75 percentile | 14 (28.6) | 29 (22.8) | 15 (31.9) | 5 (18.5) |  | >50 to 75 percentile | 3 (20.0) | 8 (29.6) | 6 (24.0) | 2 (20.0) |  |
| >75 percentile | 6 (12.2) | 22 (17.3) | 16 (34.0) | 18 (66.7) |  | >75 percentile | 0 (0.0) | 6 (22.2) | 11 (44.0) | 2 (20.0) |  |
| **16-Hydroxypalmitate** |  |  |  |  |  | **16-Hydroxypalmitate** |  |  |  |  |  |
| ≤25 percentile | 10 (20.4) | 37 (29.1) | 12 (25.5) | 3 (11.1) | 0.10 | ≤25 percentile | 5 (33.3) | 9 (33.3) | 4 (16.0) | 1 (10.0) | 0.17 |
| >25 to 50 percentile | 17 (34.7) | 27 (21.3) | 12 (25.5) | 7 (25.9) |  | >25 to 50 percentile | 2 (13.3) | 5 (18.5) | 9 (36.0) | 4 (40.0) |  |
| >50 to 75 percentile | 14 (28.6) | 28 (22.1) | 15 (31.9) | 5 (18.5) |  | >50 to 75 percentile | 6 (40.0) | 6 (22.2) | 3 (12.0) | 4 (40.0) |  |
| >75 percentile | 8 (16.3) | 35 (27.6) | 8 (17.0) | 12 (44.4) |  | >75 percentile | 2 (13.3) | 7 (25.9) | 9 (36.0) | 1 (10.0) |  |
| **Bilirubin (E,Z or Z,E)** |  |  |  |  |  | **Bilirubin (E,Z or Z,E)** |  |  |  |  |  |
| ≤27.49 percentile | 11 (22.5) | 47 (37.0) | 10 (21.3) | 1 (3.7) | 0.0024 | ≤25 percentile | 6 (40.0) | 7 (25.9) | 3 (12.0) | 3 (30.0) | 0.65 |
| >27.49 to 50 percentile | 16 (32.7) | 21 (16.5) | 13 (27.7) | 6 (22.2) |  | >25 to 50 percentile | 5 (33.3) | 5 (18.5) | 7 (28.0) | 2 (20.0) |  |
| >50 to 75 percentile | 9 (18.4) | 32 (25.2) | 15 (31.9) | 7 (25.9) |  | >50 to 75 percentile | 2 (13.3) | 8 (29.6) | 7 (28.0) | 3 (30.0) |  |
| >75 percentile | 13 (26.5) | 27 (21.3) | 9 (19.2) | 13 (48.2) |  | >75 percentile | 2 (13.3) | 7 (25.9) | 8 (32.0) | 2 (20.0) |  |
| **Cyclo (-Leu-Pro)** |  |  |  |  |  | **Cyclo (-Leu-Pro)** |  |  |  |  |  |
| ≤51.39 percentile | 33 (67.4) | 66 (52.0) | 21 (44.7) | 9 (33.3) | 0.045 | ≤25.38 percentile | 7 (46.7) | 8 (29.6) | 4 (16.0) | 0 (0.0) | 0.12 |
| >51.39 to 75 percentile | 8 (16.3) | 34 (26.8) | 11 (23.4) | 6 (22.2) |  | >25.38 to 50 percentile | 4 (26.7) | 8 (29.6) | 5 (20.0) | 3 (30.0) |  |
| >75 percentile | 8 (16.3) | 27 (21.3) | 15 (31.9) | 12 (44.4) |  | >50 to 75 percentile | 3 (20.0) | 4 (14.8) | 9 (36.0) | 2 (20.0) |  |
| - | - | - | - | - |  | >75 percentile | 1 (6.7) | 7 (25.9) | 7 (28.0) | 5 (50.0) |  |
| **Dihomo-linoleate (20:2n6)** |  |  |  |  |  | **Dihomo-linoleate (20:2n6)** |  |  |  |  |  |
| ≤25 percentile | 10 (20.4) | 32 (25.2) | 17 (36.2) | 3 (11.1) | 0.20 | ≤25 percentile | 5 (33.3) | 7 (25.9) | 6 (24.0) | 1 (10.0) | 0.90 |
| >25 to 50 percentile | 18 (36.7) | 27 (21.3) | 10 (21.3) | 5 (18.5) |  | >25 to 50 percentile | 4 (26.7) | 6 (22.2) | 5 (20.0) | 4 (40.0) |  |
| >50 to 75 percentile | 10 (20.4) | 35 (27.6) | 10 (21.3) | 11 (40.7) |  | >50 to 75 percentile | 4 (26.7) | 8 (29.6) | 6 (24.0) | 2 (20.0) |  |
| >75 percentile | 11 (22.5) | 33 (26.0) | 10 (21.3) | 8 (29.6) |  | >75 percentile | 2 (13.3) | 6 (22.2) | 8 (32.0) | 3 (30.0) |  |
| **Palmitoleate (16:1n7)** |  |  |  |  |  | **Palmitoleate (16:1n7)** |  |  |  |  |  |
| ≤25 percentile | 11 (22.5) | 33 (26.0) | 15 (31.9) | 3 (11.1) | 0.47 | ≤25 percentile | 4 (26.7) | 5 (18.5) | 8 (32.0) | 2 (20.0) | 0.46 |
| >25 to 50 percentile | 14 (28.6) | 26 (20.5) | 10 (21.3) | 6 (22.2) |  | >25 to 50 percentile | 3 (20.0) | 10 (37.0) | 4 (16.0) | 2 (20.0) |  |
| >50 to 75 percentile | 16 (32.7) | 32 (25.2) | 11 (23.4) | 10 (37.0) |  | >50 to 75 percentile | 6 (40.0) | 5 (18.5) | 4 (16.0) | 4 (40.0) |  |
| >75 percentile | 8 (16.3) | 36 (28.4) | 11 (23.4) | 8 (29.6) |  | >75 percentile | 2 (13.3) | 7 (25.9) | 9 (36.0) | 2 (20.0) |  |

* May not sum to total due to missing values.
