# Supplementary material for: Alcohol Consumption-Related Metabolites in Relation to Colorectal Cancer and Adenoma: Two Case-Control Studies Using Serum Biomarkers
Source: PLoS One. 2016 Mar 11;11(3):e0150962. doi: 10.1371/journal.pone.0150962 (PMC4788441; doi:10.1371/journal.pone.0150962)
Supplement: S2 Table — (DOCX) [file pone.0150962.s002.docx]

| **S2 Table. Pearson’s Correlation Matrix for Alcohol Consumption-Related Metabolites in 502 US Adults (PLCO) and 197 US Adults (Navy Colon Adenoma Study)** | | | | | | | | |
| --- | --- | --- | --- | --- | --- | --- | --- | --- |
| Metabolite | Ethyl Glucuronide | 4-androstene-3beta,17beta-diol disulfate 1 | 5-alpha-androstan-3beta,17beta-diol disulfate | 16-hydroxypalmitate | Bilirubin (E,Z or Z,E) | Cyclo (-leu-pro) | Dihomo-linoleate (20:2n6) | Palmitoleate (16:1n7) |
|  | rho (*P* value) | rho (*P* value) | rho (*P* value) | rho (*P* value) | rho (*P* value) | rho (*P* value) | rho (*P* value) | rho (*P* value) |
| **PLCO** |  | | | | | | | |
| Ethyl Glucuronide | 1 | 0.20 (*P* < 0.0010) | 0.12 (*P* = 0.0074) | 0.13 (*P* = 0.0027) | 0.14 (*P* = 0.0022) | 0.17 (*P* < 0.0010) | 0.14 (*P* = 0.0011) | 0.13 (*P* = 0.0034) |
| 4-androstene-3beta,17beta-diol disulfate 1 |  | 1 | 0.80 (*P* < 0.0010) | 0.20 (*P* < 0.0010) | 0.17 (*P* < 0.0010) | 0.26 (*P* < 0.0010) | 0.13 (*P* = 0.0031) | 0.054 (*P* = 0.23) |
| 5-alpha-androstan-3beta,17beta-diol disulfate |  |  | 1 | 0.15 (*P* < 0.0010) | 0.26 (*P* < 0.0010) | 0.16 (*P* < 0.0010) | 0.086 (*P* = 0.055) | -0.019 (*P* = 0.68) |
| 16-hydroxypalmitate |  |  |  | 1 | 0.45 (*P* < 0.0010) | 0.051 (*P* = 0.25) | 0.74 (*P* < 0.0010) | 0.71 (*P* < 0.0010) |
| Bilirubin (E,Z or Z,E) |  |  |  |  | 1 | 0.064 (*P* = 0.15) | 0.48 (*P* < 0.0010) | 0.43 (*P* < 0.0010) |
| Cyclo (-leu-pro) |  |  |  |  |  | 1 | 0.043 (*P* = 0.34) | 0.061 (*P* = 0.17) |
| Dihomo-linoleate (20:2n6) |  |  |  |  |  |  | 1 | 0.89 (*P* < 0.0010) |
| Palmitoleate (16:1n7) |  |  |  |  |  |  |  | 1 |
| **Navy Colon Adenoma Study** |  | | | | | | | |
| Ethyl Glucuronide | 1 | 0.36 (*P* < 0.0010) | 0.43 (*P* < 0.0010) | 0.076 (*P* = 0.29) | -0.031 (*P* = 0.67) | 0.18 (*P* = 0.012) | 0.17 (*P* = 0.020) | 0.14 (*P* = 0.045) |
| 4-androstene-3beta,17beta-diol disulfate 1 |  | 1 | 0.76 (*P* < 0.0010) | 0.22 (*P* = 0.0021) | 0.06 (*P* = 0.37) | 0.18 (*P* = 0.010) | 0.21 (*P* = 0.0027) | 0.24 (*P* < 0.0010) |
| 5-alpha-androstan-3beta,17beta-diol disulfate |  |  | 1 | 0.20 (*P* = 0.0045) | 0.076 (*P* = 0.29) | 0.14 (*P* = 0.052) | 0.23 (*P* = 0.0011) | 0.20 (*P* = 0.0053) |
| 16-hydroxypalmitate |  |  |  | 1 | 0.37 (*P* < 0.0010) | 0.22 (*P* = 0.0016) | 0.73 (*P* < 0.0010) | 0.81 (*P* < 0.0010) |
| Bilirubin (E,Z or Z,E) |  |  |  |  | 1 | -0.10 (*P* = 0.15) | 0.37 (*P* < 0.0010) | 0.40 *(P* < 0.0010) |
| Cyclo (-leu-pro) |  |  |  |  |  | 1 | 0.16 (*P* = 0.030) | 0.24 (*P* < 0.0010) |
| Dihomo-linoleate (20:2n6) |  |  |  |  |  |  | 1 | 0.87 (*P* < 0.0010) |
| Palmitoleate (16:1n7) |  |  |  |  |  |  |  | 1 |
