# Supplementary material for: Alcohol Consumption-Related Metabolites in Relation to Colorectal Cancer and Adenoma: Two Case-Control Studies Using Serum Biomarkers
Source: PLoS One. 2016 Mar 11;11(3):e0150962. doi: 10.1371/journal.pone.0150962 (PMC4788441; doi:10.1371/journal.pone.0150962)
Supplement: S3 Table — Abbreviations: CI, Confidence Interval; OR, Odds Ratio. † Adjusted for age (continuous), sex (binary: female vs. male), and tobacco smoking (categorical: current tobacco user, former tobacco user, never tobacco user, missing tobacco data). a Principal components were scaled by dividing by half the interquartile range and were then analyzed together in an unconditional multivariable logistic regression model, adjusting for age, sex, and tobacco use. (DOCX) [file pone.0150962.s003.docx]

| **S3 Table. Adjusted Odds Ratios and 95% Confidence Intervals for the Association Between Principal Components Extracted From Principal Component Analysis and fit in a Logistic Regression Model for Colorectal Cancer in 502 US Adults (PLCO) and Colorectal Adenoma in 197 US Adults (Navy Colon Adenoma Study)** | | | | |
| --- | --- | --- | --- | --- |
| Study | Principal Component^a^ | | | Global P-Value (3 df test) |
|  |  |  |  |  |
| **PLCO** | Component 1 | Component 2 | Component 3 |  |
| OR^†^ (95% CI) | 0.78 (0.48-1.26) | 1.11 (0.57-2.14) | 1.20 (0.70-2.06) | 0.67 |
| **Navy Colon Adenoma Study** | Component 1 | Component 2 | Component 3 |  |
| OR^†^ (95% CI) | 0.42 (0.17-1.03) | 1.22 (0.31-4.74) | 0.24 (0.06-0.94) | 0.052 |

Abbreviations: CI, Confidence Interval; OR, Odds Ratio

^†^ Adjusted for age (continuous), sex (binary: female vs. male), and tobacco smoking (categorical: current tobacco user, former tobacco user, never tobacco user, missing tobacco data).

^a^ Principal components were scaled by dividing by half the interquartile range and were then analyzed together in an unconditional multivariable logistic regression model, adjusting for age, sex, and tobacco use.
